# Real-World Evidence in Cost-Effectiveness Analysis of Enhanced Influenza Vaccines in Adults ≥ 65 Years of Age: Literature Review and Expert Opinion

**DOI:** 10.3390/vaccines11061089

**Published:** 2023-06-11

**Authors:** Maarten Postma, David Fisman, Norberto Giglio, Sergio Márquez-Peláez, Van Hung Nguyen, Andrea Pugliese, Jesús Ruiz-Aragón, Analia Urueña, Joaquin Mould-Quevedo

**Affiliations:** 1 Department of Health Sciences, University Medical Center Groningen, University of Groningen, P.O. Box 72, 9700 AB Groningen, The Netherlands; m.j.postma@rug.nl; 2 Department of Economics, Econometrics & Finance, Faculty of Economics & Business, University of Groningen, 9713 AB Groningen, The Netherlands; 3 Centre of Excellence in Higher Education for Pharmaceutical Care Innovation, Universitas Padjadjaran, 40132 Bandung, Indonesia; 4 Dalla Lana School of Public Health, Toronto, ON M5T 3M7, Canada; david.fisman@utoronto.ca; 5 Hospital de Niños Ricardo Gutièrrez, Buenos Aires 1425, Argentina; ngiglio@buenosaires.gob.ar; 6 Department of Economics, Economic Analysis, Faculty of Business Pablo de Olavide University, 41013 Seville, Spain; smarpel@upo.es; 7 VNH Consulting, Montreal, QC H2V 3L8, Canada; vhnguyen@vhnconsulting.com; 8 Department of Mathematics, University of Trento, 38123 Trento, Italy; andrea.pugliese@unitn.it; 9 Hospital de la Línea de la Concepción, 11300 Cádiz, Spain; jesusm.ruiz.sspa@juntadeandalucia.es; 10 Centro de Estudios para la Prevención y Control de Enfermedades Transmisibles, Universidad Isalud, Buenos Aires C1095AAS, Argentina; cepycet@isalud.edu.ar; 11 CSL Seqirus Inc., Summit, NJ 07901, USA

**Keywords:** influenza, enhanced vaccine, adjuvanted, cost-effectiveness analysis

## Abstract

Influenza vaccination can benefit most populations, including adults ≥ 65 years of age, who are at greater risk of influenza-related complications. In many countries, enhanced vaccines, such as adjuvanted, high-dose, and recombinant trivalent/quadrivalent influenza vaccines (aTIV/aQIV, HD-TIV/HD-QIV, and QIVr, respectively), are recommended in older populations to provide higher immunogenicity and increased relative vaccine efficacy/effectiveness (rVE) than standard-dose vaccines. This review explores how efficacy and effectiveness data from randomized controlled trials and real-world evidence (RWE) are used in economic evaluations. Findings from published cost-effectiveness analyses (CEA) on enhanced influenza vaccines for older adults are summarized, and the assumptions and approaches used in these CEA are assessed alongside discussion of the importance of RWE in CEA. Results from many CEA showed that adjuvanted and high-dose enhanced vaccines were cost-effective compared with standard vaccines, and that differences in rVE estimates and acquisition price may drive differences in cost-effectiveness estimates between enhanced vaccines. Overall, RWE and CEA provide clinical and economic rationale for enhanced vaccine use in people ≥ 65 years of age, an at-risk population with substantial burden of disease. Countries that consider RWE when making vaccine recommendations have preferentially recommended aTIV/aQIV, as well as HD-TIV/HD-QIV and QIVr, to protect older individuals.

## 1. Introduction

Each year, seasonal influenza is associated with substantial global disease burden experienced by patients, caregivers, and communities. In the United States, the cost of influenza burden is estimated at $11.2 billion annually (2015 US dollars), comprising direct medical costs, such as healthcare visits (estimated at around $3 billion), and indirect costs, such as lost days at work (estimated at around $8 billion) [1]. Preventative vaccination is a key strategy by which societies can minimize influenza-related disease burden and economic cost. The US Centers for Disease Control and Prevention (CDC) estimated that 3.7 million medical visits, 105,000 hospitalizations, and 6300 deaths related to influenza were prevented by vaccination during the 2019–2020 influenza season [2]. The overall value of influenza vaccination may be underestimated [3].

National advisory bodies, such as the US Advisory Committee on Immunization Practices (ACIP), recommend that all individuals ≥ 6 months of age receive an annual vaccine for protection against seasonal influenza [4]. The need for annual vaccination is driven by ongoing evolution of the influenza virus, owing to a segmented RNA genome, which is subject to mutation and genome reassortment [5,6]. Each year, the World Health Organization (WHO) reviews influenza surveillance data and presents recommendations to regulators and vaccine manufacturers for the composition of influenza vaccines [7]. The ongoing potential for antigenic drift and resulting mismatch between vaccine and virus necessitates continual monitoring and data collection on vaccine performance in the real world [8].

All populations can benefit from influenza vaccination, although certain groups, including older adults ≥ 65 years of age, are at greater risk of influenza-related complications [1,3]. Older individuals, who show age-related declines in immune system function [9], have disproportionately high rates of seasonal influenza-related hospitalizations and deaths [2,10]. For this population, enhanced vaccines have been designed to mitigate the effects of age-related immunosenescence by providing higher immunogenicity and increased relative vaccine efficacy/effectiveness (rVE) compared with standard vaccines [10,11,12].

Enhanced vaccines use different strategies to augment immune responses and have been available for varying lengths of time in different countries (Table 1). High-dose trivalent/quadrivalent influenza vaccines (HD-TIV/HD-QIV) contain four-times more hemagglutinin antigen than standard-dose vaccines, an approach demonstrated to increase the magnitude of the immune response [10]. Adjuvanted trivalent/quadrivalent influenza vaccines (aTIV/aQIV) contain the adjuvant MF59, an oil-in-water emulsion of squalene oil, which has been demonstrated to increase the magnitude and breadth of immune responses [13,14,15]. Recombinant quadrivalent influenza vaccines (QIVr) use a higher antigen content and recombinant technology for synthetic (non-egg, non-cell) manufacture that eliminate the risk of viral mutations, an approach that may limit antigenic mismatch [16].

Adult vaccination against seasonal influenza is recommended in many countries and some, such as several countries in Europe, provide influenza vaccination free-of-charge at the point of delivery to older individuals [17]. Many European countries recommend that older individuals receive an enhanced influenza vaccine [11]. Outside Europe, in many countries, including the United States, the United Kingdom, Australia, and Argentina, enhanced vaccines are preferentially recommended over other influenza vaccines in older populations [18,19,20,21,22]. To make recommendations, national vaccine policymakers consider many factors, such as vaccine evidence, public health priorities, cost, and the ability to implement new interventions in a timely, feasible, and sustainable manner. The findings of cost-effectiveness analyses (CEA) may also be considered when making recommendations [23], with the United Kingdom Joint Committee on Vaccination and Immunization (JCVI) stating that analysis of cost-effectiveness is the “cornerstone of decision-making” related to universal vaccination decision-making and implementation [24].

The robustness of conclusions from economic models depends on the quality, accuracy, and appropriateness of a large range of data inputs and assumptions. Randomized controlled trials (RCTs) provide a gold-standard methodology to answer specific clinical research questions [25]. However, evidence that is of interest to vaccine advisory bodies, payers, and health economists, such as effectiveness data sets from multiple seasons describing patient-centric outcomes, may exceed what can be achieved practically with RCTs, which may take years to plan, implement, and analyze, and may not produce broadly generalizable findings across influenza seasons and patient populations [25,26,27]. To supplement efficacy data from RCTs, real-world evidence (RWE) provides timely and expanding datasets to monitor and evaluate vaccine effectiveness [27,28], which is important given the dynamics of a continuously changing influenza virus [5,6]. The use of RWE is increasing over time as familiarity and acceptance of data from well-constructed studies with non-randomized designs grow, especially for evaluating vaccines [29,30].

The objective of this review is to improve understanding of how efficacy and effectiveness data from RCT and RWE sources, and other parameters, are used in economic evaluations by providing an overview of published CEA on enhanced vaccines for influenza in older adults. This paper aims to critically assess assumptions and approaches in these CEA and to discuss the importance of RWE in evaluating vaccine effectiveness (VE) against influenza, with a particular focus on rVE inputs. Expert opinion on the importance, challenges, and future directions of RWE and CEA related to influenza vaccines is provided.

This paper adds to the contributions of prior articles reviewing CEA of enhanced vaccines in older adults [31,32,33,34]. In line with best practices, meta-analysis techniques are inappropriate for summarizing the outputs of economic modeling studies [35]; however, differences between economic models, including how investigators select inputs, are of interest to discuss.

**Table 1:** Currently available enhanced vaccines for older adults.

	aTIV (Fluad, Seqirus Inc.)	aQIV (Fluad, Seqirus Inc.)	HD-TIV (Fluzone, Sanofi)	HD-QIV (Fluzone, Sanofi)	QIVr (Flublok, Sanofi)
Composition	MF59^®^-adjuvanted trivalent influenza vaccine	MF59^®^-adjuvanted quadrivalent influenza vaccine	High-dose trivalent influenza vaccine	High-dose quadrivalent influenza vaccine	Recombinant quadrivalent influenza vaccine
**Approvals in select countries**					
Argentina	**2021** Adults ≥ 65 years of age [22]	NA	**2010** Adults 18–59 years of age [36]	NA	NA
Canada	**2011** Adults ≥ 65 years of age [37]	NA	**2010** adults 18–59 years of age [36] **2020** adults ≥ 65 years of age [38]	**2021** Adults ≥ 65 years of age [38]	**2021** Adults ≥ 18 years of age [39]
United States	**2015** Adults ≥ 65 years of age [40]	**2020** Adults ≥ 65 years of age [41]	**2009** adults ≥ 65 years of age [38] **2011** adults 18–64 years of age [42]	**2019** Adults ≥ 65 years of age [42]	**2013** Adults ≥ 18 years of age [43]
United Kingdom	**2017** Adults ≥ 65 years of age [44]	**2021** Adults ≥ 65 years of age [45]	**2019** Adults ≥ 65 years of age [46]	**2021** Adults ≥ 60 years of age [47]	**2022** Adults ≥ 18 years of age [45]
European Union	**2017** Adults ≥ 65 years of age [48]	**2020** Adults ≥ 65 years of age [15]	**2009** Adults 18–59 years of age [36]	**2021** Adults ≥ 60 years of age [47]	**2020** Adults ≥ 18 years of age [49]

aQIV, adjuvanted quadrivalent influenza vaccine; aTIV, adjuvanted trivalent influenza vaccine; HD-QIV, high-dose quadrivalent influenza vaccine; HD-TIV, high-dose trivalent influenza vaccine; NA, not available; QIVr, recombinant quadrivalent influenza vaccine.

## 2. Methods

### 2.1. Targeted Literature Search

A targeted literature review was performed to identify economic evaluations of enhanced influenza vaccines (aTIV/aQIV, HD-TIV/HD-QIV, and QIVr) in older individuals. MEDLINE (PubMed) was searched for publications using the following strings in October 2022, limited to studies from the past 10 years and prioritizing English-language publications. Additional articles published through March 2023 were included based on follow up searches.

- (“Adjuvanted quadrivalent influenza vaccine” OR “Fluad” OR “aIIV4” OR “aQIV”) AND (“economic” OR “cost” OR “cost effectiveness” OR “cost utility” OR “budget impact”)
- (“Adjuvanted trivalent influenza vaccine” OR “Fluad” OR “aIIV3” OR “aTIV”) AND (“economic” OR “cost” OR “cost effectiveness” OR “cost utility” OR “budget impact”)
- (“High dose quadrivalent influenza vaccine” OR “IIV4 HD” OR “QIV HD” OR “Fluzone HD”) AND (“economic” OR “cost” OR “cost effectiveness” OR “cost utility” OR “budget impact”)
- (“High dose trivalent influenza vaccine” OR “IIV3 HD” OR “TIV HD” OR “Fluzone HD”) AND (“economic” OR “cost” OR “cost effectiveness” OR “cost utility” OR “budget impact”)
- (“Quadrivalent recombinant influenza vaccine” OR “QIVr” OR “Flublok”) AND (“economic” OR “cost” OR “cost-effectiveness” OR “cost-utility” OR “budget impact”).

### 2.2. Supplemental Searches

The reference lists of retrieved primary studies, systematic reviews, and meta-analyses were searched to capture additional studies. Congress presentations that included CEA or cost-utility analysis (CUA; hereafter referred to simply as CEA for convenience) were included based on expert knowledge and ability to retrieve poster and oral presentations.

### 2.3. Included Studies

Identified papers describing CEA from any world region were prioritized for inclusion. Included papers regarded enhanced vaccines in populations ≥ 65 years of age (or >50 years of age in models regarding QIVr). To be included, studies reported multiple parameters from the following: model type, country setting, vaccine strategy, study perspective, time horizon, selected costs, currency, rVE and/or VE, discounting strategies, and use of uncertainty analyses. Included studies could use VE and rVE inputs generated from RCTs and/or RWE.

Publications identified via the search strings, published reference lists, and based on expert knowledge are captured in Table 2 and Table 3. Systematic review methodologies were not used.

## 3. Cost-Effectiveness Studies with Enhanced Influenza Vaccines

### 3.1. Comparison between CEA for Enhanced and Standard Vaccines

In many countries, CEA have estimated the economic value of enhanced vaccines for older populations. Thirty-one CEA comparing enhanced vaccines to standard-dose vaccines were analyzed, 17 comparing aTIV/aQIV with TIV/QIV (Table 2A) and 14 comparing HD-TIV/HD-QIV with TIV/QIV (Table 2B). Studies included static and dynamic designs, and perspectives included healthcare system, societal, and third-party payer. Most studies included probabilistic and/or deterministic sensitivity analyses. Time horizons varied from one influenza season or year, although some models took a multi-year or lifetime approach. Discounting ranged from 0–5% for outcomes and costs. Most studies had an industry sponsor.

**Figure 1:**
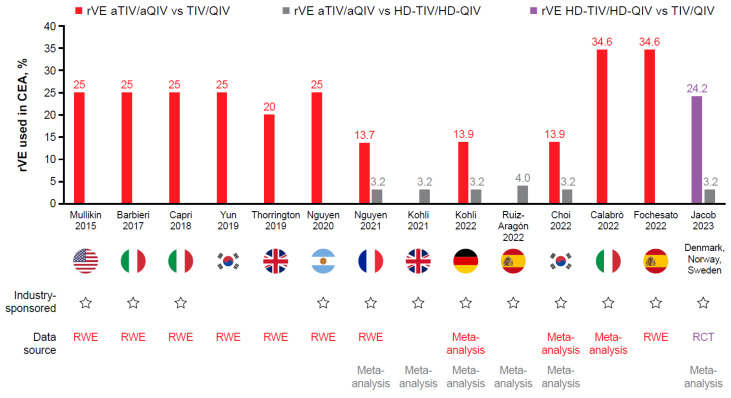
rVE as used in base-case analyses of aTIV/aQIV compared with TIV/QIV and/or HD-TIV/HD-QIV [51,52,53,54,55,56,57,58,59,60,61,62,63,64]. aQIV, adjuvanted quadrivalent influenza vaccine; aTIV, adjuvanted trivalent influenza vaccine; CEA, cost-effectiveness analysis; HD-QIV, high-dose quadrivalent influenza vaccine; HD-TIV, high-dose trivalent influenza vaccine; QIV, quadrivalent influenza vaccine; RCT, randomized controlled trial; rVE, relative vaccine effectiveness; RWE, real-world evidence; TIV, trivalent influenza vaccine.

**Figure 2:**
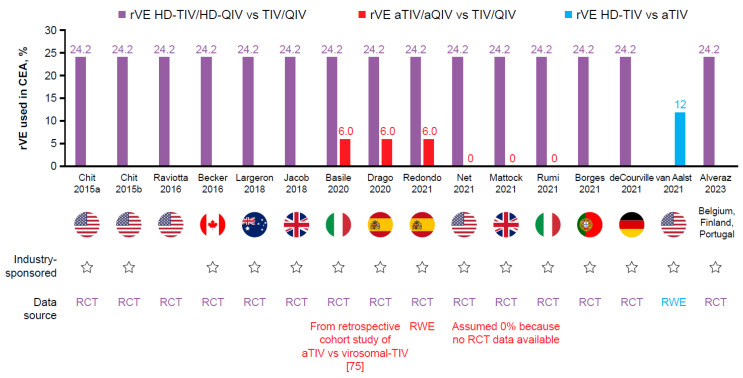
rVE as used in base-case analyses of HD-TIV/HD-QIV compared with TIV/QIV or aTIV/aQIV [65,66,67,68,69,70,71,72,73,74,75,76,77,78,79,80,81]. Note: no value indicates that a comparison was not evaluated. 0 indicates a rVE of 0% in base-case analysis. Note: rVE aTIV/aQIV versus TIV/QIV is captured as reported in studies that use a common comparator of TIV/QIV to indirectly compare rVE of HD-TIV/HD-QIV versus aTIV/aQIV. aQIV, adjuvanted quadrivalent influenza vaccine; aTIV, adjuvanted trivalent influenza vaccine; CEA, cost-effectiveness analysis; HD-QIV, high-dose quadrivalent influenza vaccine; HD-TIV, high-dose trivalent influenza vaccine; QIV, quadrivalent influenza vaccine; RCT, randomized controlled trial; rVE, relative vaccine effectiveness; RWE, real-world evidence; TIV, trivalent influenza vaccine.

Inputted rVE values varied across studies. In studies of adjuvanted versus standard-dose vaccines, estimates of rVE for aTIV/aQIV versus TIV/QIV ranged from 13.7% to 34.6%, which represents RWE estimates of rVE against laboratory-confirmed influenza (LCI), hospitalization/healthcare visits, or other measures (Table 2A; Figure 1). The rVE of aTIV/aQIV versus TIV/QIV was also captured as reported in studies that used a common comparator of TIV/QIV to indirectly compare rVE of HD-TIV/HD-QIV versus aTIV/aQIV. Interestingly, in this case, much lower estimates of rVE, ranging from 0% to 6% for aTIV/aQIV versus TIV, were input into CEA models (Table 3B; Figure 2). The rVE estimate of 24.2% for HD-TIV/HD-QIV versus TIV/QIV for LCI cases, based on results from the FIM12 RCT [50], was consistently used in CEA comparing HD-TIV/HD-QIV versus TIV/QIV, and used in CEA that indirectly compared the rVE of HD-TIV/HD-QIV versus aTIV/aQIV (Table 2B and Table 3B; Figure 2).

**Table 2:** Cost-effectiveness studies of adjuvanted vaccines (**A**) or high-dose vaccines (**B**), compared with TIV/QIV.

Author Year	Country	Strategy	Model Type	Perspective	Time Horizon	Selected Costs	Year, Currency	rVE *	Discounting	Uncertainty Analysis	Findings	Author Conclusion	Industry Sponsor
**(A) Adjuvanted vaccines vs. TIV/QIV**													
Lee BY, et al., 2009 [82]	USA	aTIV vs. TIV	Decision analytic computer simulation model	Societal, third-party payer	1 influenza season	TIV $15.75 (price obtained from Red Book) aTIV varied $0–100 to that of TIV Hospitalization Death Complications Medical visits Lost productivity	2007, US$	aTIV potency 50% (ability to overcome immunosenescence; origin of estimate undisclosed)	NR	Univariate, multi- dimensional, PSA	aTIV vs. TIV could prevent: 496,533 influenza cases 171,981 hospitalizations 70,429 deaths Save society $824 million if aTIV cost the same as TIV (dominant), and continue to be cost-saving if aTIV cost $30 more than TIV	Introducing aTIV to older adults could save significant morbidity, mortality, and costs. aTIV remained a dominant strategy in several scenarios	No
Fisman DN and Tuite AR 2011 [83]	Canada	aTIV vs. TIV	Age-structured compartmental model	NR	10 years	TIV CAN$7.55 aTIV CAN$11.59 (from literature; type of price undisclosed) Influenza infection Hospitalization ICU admission ED visit GP visit Death	2009, CAN$	VE aTIV 40% VE TIV 20% (multiple RWE sources used for model calibration, including meta-analysis by Jefferson [84])	Costs 5% QALYs lost 5%	One-way, Multivariate	aTIV cost more vs. TIV, but cost was offset by fewer influenza cases and decreased healthcare resource use from CAN$501.76 million to CAN$473.50 million ICER $2111/QALY	aTIV in adults ≥ 65 years of age was highly cost-effective vs. TIV	Yes (Novartis)
Mullikin M, et al., 2015 [51]	USA	aTIV vs. TIV and QIV	Compartmental, dynamic epidemiologic module (SIR model) and tree-structured outcomes model	NR	1 year	TIV $9.45 aTIV $13.65 QIV $13.65 (price assumed, or from CDC) Hospitalization Death Complications Medical visits Comedication Lost productivity Administration	NA, US$	rVE aTIV vs. TIV 25% any strain (from prospective, observational study [85])	Costs 3% Life-years and QALYs lost 3%	Univariate, PSA	aTIV vs. TIV in persons ≥ 65 years of age: ICER $9980–28,800/QALY aTIV vs. QIV in persons ≥ 65 years of age: dominant	aTIV in adults ≥ 65 years of age may enable clinical and economic benefit vs. QIV and TIV	Yes (Novartis)
Ruiz-Aragón J, et al., 2015 [86]	Spain	aTIV vs. TIV	Scenario-based budget impact analysis ^†^	NR	NR	TIV €3.75 aTIV €4.30 (weighted average of the prices extracted from the contract of tender for the 2012–2013 campaign of the Andalusian Service of Health) Medical consultation Hospitalization Comedication	NA, Euro €	rVE, NR	NR	Univariate	113,189 influenza cases were avoided €79.99 million was saved, leading to a budget impact of €76.13 million saved	Adding aTIV to those > 64 years of age would provide significant savings for the health system (article in Spanish)	No
Barbieri M and Capri S 2017 [52]	Italy	aTIV vs. TIV. QIV, ID-TIV, no vaccination	Decision tree model	NR	NR	aTIV €6.99 TIV €5.35 ID-TIV €6.99 QIV €11.08 (ex-factory prices) Hospitalization Medical visits Death Complications	NR, Euro €	rVE aTIV vs. TIV 25% (from prospective, observational study [85]) ID-TIV vs. TIV: 16.5% (from modeled data [87]) VE TIV 58% (from meta-analysis [84])	NR	Univariate, PSA	aTIV vs. TIV ICER €4527/QALY aTIV dominated ID-TIV aTIV dominated QIV aTIV vs. no vaccination ICER €10,750/QALY	aTIV should be the vaccine of choice for older adults ≥ 65 years of age in Italy and is cost-effective vs. TIV and no vaccination (article in Italian)	No
Pérez-Rubio A and Eiros JM 2018 [88]	Spain	aTIV vs. TIV	Scenario-based budget impact analysis ^†^	NR	NR	TIV €2.90 aTIV €4.30 (public data) Medical consultation Comedication	NA, Euro €	rVE, not available	NR	Univariate	Budgetary impact of replacing TIV with aTIV was €6.97 million, suggesting a potential saving of €82 million Cost–benefit ratio of 12.83	Replacing TIV with aTIV in those ≥ 65 years of age would increase the efficiency of the vaccination programs in Spain and its autonomous communities (article in Spanish)	Seqirus acknowledged
Capri S, et al., 2018 [53]	Italy	aTIV vs. TIV, ID-TIV, QIV	Decision tree model	Italian NHS	1 year	TIV €5.35 aTIV €6.99 ID-TIV €6.99 QIV €11.08 (ex-factory price; public data) Medical consultation Comedication Complications	2017, Euro €	VE TIV 58% (from meta-analysis [84]) rVE aTIV vs. TIV 25% (from prospective, observational study [85]) ID-TIV vs. TIV: 16.5% (from modeled data [87]) rVE QIV vs. TIV 3.8% (estimated)	Costs 0% Loss of QALYs discounted	One-way, DSA, PSA	aTIV vs. TIV ICER €4527/QALY aTIV dominated ID-TIV and QIV	aTIV should be preferred for Italians ≥ 65 years of age	Yes (Seqirus)
Yun JW, et al., 2019 [54]	South Korea	aTIV vs. TIV QIV vs. TIV	Static lifetime Markov model Analyzed across three age groups (65–74, 75–84, and ≥85 years of age)	Societal	Lifetime	TIV $7.47 QIV $8.59 aTIV $8.59 (purchase price of NIP or assumed) Administration Hospitalization Medical visits Death Complications	2016, US$	VE aTIV 60.30% (calculated from prospective, observational study [85]) VE TIV 48.24%, VE QIV 57–58% (calculated from several meta-analyses [84,89,90])	Costs 3% Outcomes 3%	One-way, PSA	Compared with TIV, aTIV reduced: cases by 1,812,395 and complications by 89,747 aTIV was highly cost-saving and dominated TIV QIV vs. TIV ICER $17,699/QALY	aTIV and QIV were more cost-effective than TIV for those ≥ 65 years of age	No
Thorrington D, et al., 2019 [55]	England	aTIV vs. TIV	Dynamic SEIR-type transmission model with economic framework in adults ≥ 65 and ≥75 years of age	Healthcare provider	14 seasons used in model	£11.75 aTIV £9.05 TIV (list price including VAT) GP consultation Hospitalization	NR, GBP£	rVE aTIV vs. TIV 20% (assumption, designed to be more conservative than community-based case–control study [91])	Costs adjusted for inflation	DSA, PSA	Compared with TIV, aTIV reduced: GP consultations by 18,913, hospitalizations by 1152, and deaths by 380 aTIV vs. TIV ICER £469/QALY	Compared with TIV, aTIV reduced healthcare use and was more cost-effective in persons ≥ 65 years of age Persons ≥ 75 years of age may receive the greatest benefit from aTIV given the lack of efficacy of TIV in this age group	No
Nguyen VH, et al., 2020 [56]	Argentina	aTIV vs. TIV	Decision tree model	Payer	1 year	TIV $4.73 (public price) aTIV $7.00 (list price) Hospitalization Outpatient care Administration Consultation Drug/antivirals	NR, US$	rVE aTIV vs. TIV 25% (from prospective, observational study [85])	Costs 0% Outcomes 0%	Univariate DSA, PSA	Compared with TIV, switching to aTIV could reduce: cases by 20,930, GP visits by 15,120, hospitalizations by 530, deaths by 170, and life years lost by 1640 Gain 1310 QALYs aTIV vs. TIV ICER $2660.59/QALY	aTIV yielded substantial health benefits and cost savings vs. TIV in older adults. rVE and influenza attack rate were most influential in DSA.	Yes (Seqirus)
Nguyen VH, et al., 2021 [57]	France	aQIV vs. QIVe aQIV vs. HD-QIV	Static decision tree model	Payer	NR	QIV €11.11 aQIV €26.00 HD-QIV €26.00 (assumption) Healthcare visit In/outpatient complications Hospitalization Mortality	NR, Euro €	rVE aTIV vs. QIV 13.7% (95% CI 3.1, 24.2) * rVE aTIV vs. HD-TIV 3.2% (−2.5, 8.9) * rVE aTIV vs. TIV 13.9% (4.2, 23.5) * (from meta-analysis [92])	NR	DSA	Replacing QIVe with aQIV over a 3-year period could prevent: 56,028 influenza cases, 13,449 medical care visits, 30,815 outpatient complications, 3902 inpatient complications, and 745 influenza-associated deaths Budget savings were driven by avoidance of medical care visits costs (€470 K); outpatient complication costs (€788 K) and inpatient complication costs (€23.2 M).	aQIV for the older adult population would be clinically favorable, with a small incremental cost impact	Yes (Seqirus)
Angerami R, et al., 2021 [93]	Brazil	aTIV vs. TIVe	Static decision tree model based on epidemiology and demography across 10 seasons	Societal, payer	1 year	TIVe R$15.12 aTIV R$27.65 (list prices with or without adjustment) Medical visit Hospitalization Absenteeism Death	NR, Brazilian Reais R$	rVE assumed from Italian multi-season analysis (value not stated)	NR	PSA	Compared with TIVe, aTIV reduced: cases by 300,035, outpatient visits by 90,589, hospitalizations by 23,100, and deaths by 4931 QALYs increased by 49,457 aTIV vs. TIVe ICER R$6253/QALY (payer perspective)	aTIV was highly cost-effective compared with TIVe	Yes (Seqirus)
Kohli M, et al., 2022 [58]	Germany	aQIV vs. QIVe aQIV vs. HD-QIV	SEIR compartmental transmission model	Societal, Statutory health insurance	10 seasons from 2010–2019	QIVe €12.56 aQIV €19.21 HD-QIV €40.55 (reimbursement price per dose) Hospitalization Death In/outpatient visits Medication Sickness benefit Lost working time	NA, Euro €	aQIV vs. QIVe 13.9% (4.2, 23.5) * aQIV vs. HD-QIV 3.2% (−2.5, 8.9) * (from meta-analysis [92]) VE QIVe 62%, 24%, and 79% against A/H1N1, A/H3N2, and B types (assumptions, related to meta-analysis [94] and systematic review [90])	Costs 3% QALYs 3%	DSA, PSA	aQIV and HD-QIV reduced the number of influenza cases, hospitalizations, and deaths in the German population vs. QIVe. aQIV dominated HD-QIV because it was slightly more effective in the base case (rVE = 3.2%), and was less costly to implement	aQIV may be cost-effective compared with QIVe at current prices aQIV and HD-QIV had similar clinical effectiveness, but aQIV is less costly than HD-QIV. CE of aQIV was most sensitive to changes in VE and rate of hospitalization due to influenza	Yes (Seqirus)
Choi MJ, et al., 2022 [59]	South Korea	aQIV vs. QIV aQIV vs. HD-QIV	Static, 1-year decision tree model Analyzed across three age groups (65–74, 75–84, and ≥85 years of age)	Healthcare system	1 year	Hospitalization Death Complications Influenza cases Vaccine price NR	NR	aQIV vs. QIVe 13.9% (4.2, 23.5) * aQIV vs. HD-QIV 3.2% (−2.5, 8.9) * (from meta-analysis [92]) VE QIV 62%, 24%, and 63% vs. A(H1N1), A(H3N2), and B, respectively (from meta-analysis [94])	NR	DSA, PSA	Compared with QIV, aQIV reduced: cases by 35,390, complications by 1602, hospitalizations by 709, and deaths by 145 Compared with HD-QIV, aQIV reduced: cases by 7247, complications by 328, hospitalizations by 145, and deaths by 30	Replacing QIV with aQIV is predicted to reduce disease burden in South Korean adults ≥ 65 years of age Benefits of aQIV and HD-QIV are predicted to be similar due to comparable VE CE estimates were most influenced by changes to rVE	Yes (Seqirus)
Calabrò GE, et al., 2022 [60]	Italy	aQIV vs. QIVe	SEIR dynamic transmission model	Societal, health system payer	Nine seasons	Infection Hospitalization Death Medical visits Complications Vaccine price NR	2020, Euro €	rVE aTIV vs. TIVe or QIVe 34.6% (2.0, 66.0) LCI* (estimated based on data from meta-analysis [in Italian])	Indirect costs 3% QALYs 3% Costs inflated to 2020	DSA, PSA	Across all age categories, aQIV could avoid 363 hospitalizations and 195 deaths vs. QIVe—of these, 93% of avoided hospitalizations and 98% of avoided deaths would be recorded in those > 65 years of age aQIV vs. QIVe ICER: €14,441/QALY	aQIV in individuals ≥ 65 years of age is cost-effective	Yes (Seqirus)
Fochesato A, et al., 2022 [61]	Spain	aQIV vs. QIVe	SEIR dynamic transmission model	Societal, public payer	Cost time horizon = one season Effect time horizon = lifetime	aQIV €13.00 QIVe €9.50 (per dose, unspecified) Disease management Hospitalization Medical visits Vaccines Loss of productivity Death	2021, Euro €	rVE aTIV vs. TIVe or QIVe 34.6% (2.0, 66.0) LCI* (estimated based on data from meta-analysis [in Italian]) rVE aQIV vs. QIVe 13.9% (4.2, 23.5) * (from meta-analysis [92]) VE QIVe 62%, 24%, and 52.1% vs. A(H1N1), A(H3N2), and B, respectively (taken from secondary sources [in Italian] including [95]	Costs 3% QALY 3%	DSA, PSA	aQIV vs. QIVe with rVE 34.6% reduced: cases by 43,664, hospitalizations by 1111, and deaths by 569 aQIV vs. QIVe with rVE 13.9% reduced: cases by 19,104, hospitalizations by 486, and deaths by 252 ICER €2240/QALY for rVE 34.6% ICER €6694/QALY for rVE 13.9% (payer perspective)	Replacing QIVe with aQIV when vaccinating adults ≥ 65 years of age in Spain is a cost-effective strategy in high and moderate rVE scenarios	Yes (Seqirus)
Jacob J, et al., 2023 [62]	Denmark, Norway, Sweden	aQIV vs. QIV	Static decision tree model	Healthcare payer, societal	NR	QIV €9.10–11.00 aQIV 170–189% that of QIV (prices from IQVIA or assumption) Hospitalization GP visit Outpatient visit Comedication Lost productivity Death Complications Influenza cases	2022, Euro €	VE QIV 62%, 24%, and 63% vs. A(H1N1), A(H3N2), and B, respectively (from meta-analysis [94]) rVE HD-QIV to QIV 24.2% * from FIM12 RCT [50]	3–4% outcomes and costs	DSA, PSA	Across Denmark, Norway, and Sweden in one influenza season, aQIV vs. QIV could prevent: 18,772 symptomatic influenza infections, 925 hospitalizations, and 161 deaths aQIV vs. QIV ICER €10,170/QALY in Denmark ICER €12,515/QALY in Norway ICER €9894/QALY in Sweden	Introducing aQIV to those ≥ 65 years of age may reduce influenza disease and economic burden in Denmark, Norway, and Sweden	Yes (Seqirus)
**(B) High-dose vaccines vs. TIV/QIV**													
Chit A, et al., 2015a [65]	USA	HD-TIV vs. TIV	CEA, person-level study	Societal Third-party payer	Cost = one influenza season Effect = lifetime	HD-TIV $31.82 TIV $12.04 (unit costs) Hospitalization Deaths Medical visits Prescription medication Study vaccine Lost work force	NR, USD$	rVE HD-TIV vs. TIV 24.2% from FIM12 RCT [50]	NR	PSA	Societal and Medicare perspectives: HD-TIV dominated TIV Mean per-participant medical costs were lower with HD-TIV ($1376.72) than TIV ($1492.64)Hospital admissions contributed 95% of the total healthcare-payer cost and 87% of the total societal costs	HD-TIV is less costly and more effective vs. TIV, driven by a reduction in the number of hospital admissions PSA showed HD-TIV 93% likely to be cost-saving	Yes (Sanofi)
Chit A, et al., 2015b [66]	USA	HD-TIV vs. TIV HD-TIV vs. QIV	Economic model evaluating three health states: symptomatic influenza, influenza-associated hospitalizations, and influenza-associated deaths	Societal, Third-party payer	Cost time horizon = one influenza season Effect time horizon = lifetime	HD-TIV $32.82 TIV $12.39 QIV $19.41 (CMS costs per dose) Symptomatic influenza Hospitalization Medical visits Comedication Work loss Co-payments	NR, USD$	rVE HD-TIV vs. TIV 24.24% (9.69, 36.52) symptomatic influenza from FIM12 RCT [50] VE TIV 49% (33.00, 62.00) symptomatic influenza (from meta-analysis [96]) VE QIV 50.68% (34.13, 64.13) symptomatic influenza (estimated based on multiple sources including from meta-analysis [96])	Costs 0% Outcomes 3%	DSA, PSA	Compared with TIV, HD-TIV could avoid 195,958 cases of influenza, 22,567 influenza-related hospitalizations, and 5423 influenza-related deaths Compared with QIV, HD-TIV could avoid 169,257 cases of influenza, 21,222 hospitalizations, and 5212 deaths Societal: HD-TIV vs. TIV ICER $5299/QALY HD-TIV dominated QIV Third-party payer: HD-TIV vs. TIV ICER $10,350/QALY HD-TIV vs. QIV ICER $4365	HD-TIV is expected to be cost-effective vs. TIV and QIV. 60–71% probability HD-TIV is at least cost-effective compared with TIV. 70–81% probability HD-TIV is at least cost-effective compared with QIV	Yes (Sanofi)
Cheng X and Roïz J 2015 [97]	Canada	HD-TIV vs. TIV	Analytical decision model	Healthcare, societal	NR	Comedication Long-term impact of influenza infections Vaccine price NR	NR, CAN$	NR	Costs NR Outcomes NR	DSA, PSA	HD-TIV vs. TIV ICER CAN$3763/QALY healthcare perspective ICER CAN$190/QALY societal perspective HD-TIV dominated TIV when long-term care costs were considered	HD-TIV may reduce influenza-associated morbidity and mortality, and is cost-effective in the studied population vs. TIV	No
Becker D, et al., 2016 [67]	Canada	HD-TIV vs. TIV	CEA, person-level study	Societal Public health payer	Cost time horizon = one influenza season Effect time horizon = lifetime	HD-TIV: $31.82 TIV: $5.82 (CMS price schedule and manufacturer) ER visits Hospitalization Medical visits Comedication Lost work force	2014, CAN$	rVE HD-TIV vs. TIV 24.2% (9.7, 36.5) LCI from FIM12 RCT [50]	Costs 0% Outcomes 5%	PSA	HD-TIV dominated TIV from public payer and societal perspective Per-participant total societal costs were were lower with HD-TIV (CAN$814) than TIV (CAN$874). 91% of healthcare payer costs and 76% of the total societal costs were due to hospital admissions	HD-TIV is expected to be a less costly and more effective vs. TIV driven by a reduction in hospitalizations PSA indicated HD-TIV is 89% likely to be cost-saving	Yes (Sanofi)
Raviotta J, et al., 2016 [68]	USA	HD-TIV vs. QIV	Markov state transition model	Societal	Cost time horizon = one influenza season Effect time horizon = lifetime	HD-TIV: $31.20 TIV: $10.69 QIV $16.15 (CMS price schedule and medical literature) Hospitalization Influenza illness Death Outpatient Medication Vaccine Productivity loss	2014 USD$	VE all vaccines 39% (from modeled US data [98]) rVE HD-TIV vs. TIV: 24.2% * from FIM12 RCT [50]	Costs 0% Outcomes 3%	One-way, PSA	HD-TIV vs. QIV ICER $31,214/QALY. Despite a substantially higher per-dose cost ($21.51 more), HD-TIV is an economically favorable strategy in for US adults ≥ 65 years of age Secondary analysis: aTIV was not favored vs. TIV if rVE was < 15% but was favored if rVE aTIV vs. TIV ≥ 32%. If rVE was equivalent to that of HD-TIV (i.e., 24.2%), it would be favored if it cost less than HD-TIV	HD-TIV for adults ≥ 65 years of age is likely to be favored from economic and public health standpoints. Results were sensitive to yearly influenza attack rates, virus variability, and VE	No
Crépey P, et al. 2018 [99]	England and Wales	HD-TIV vs. TIV	Dynamic compartmental transmission model	NR	Cost time horizon = 8 years Effect time horizon = 8 years	Hospitalization Influenza cases GP consultations Death Vaccine price NR	NR, GBP£	rVE from FIM12 RCT [50] (specific value NR in abstract)	Costs NR Outcomes NR	PSA	In an average season, HD-TIV rather than TIV could prevent: 8500 GP consultations, 800 influenza-related hospitalizations, and 600 deaths HD-TIV economically justifiable prices of £27.00 and £36.80 per dose for ICER thresholds of £20,000/QALY and £30,000/QALY, respectively; higher prices were justifiable when accounting for the vaccine impact on cardiorespiratory events	Vaccination of adults ≥ 65 years of age with HD-TIV in the UK is likely to be a highly cost-effective vs. TIV. This benefit is driven by a reduction in influenza-related hospitalizations	Yes (Sanofi)
Jacob J, et al., 2018 [69]	England and Wales	HD-TIV vs. TIV	Age-structured decision tree model	Public healthcare payer	1 year, with longer time horizon for QALYs	Hospitalization Influenza cases GP consultations Death Vaccine list price	2017, GBP£	rVE HD-TIV vs. TIV 24.2% from FIM12 RCT [50]	Costs 0% Outcomes 3.5%	DSA	In an average season, HD-TIV rather than TIV could prevent: 75,000 cases of confirmed influenza, 19,000 influenza-related hospitalizations, and 4000 deaths Using thresholds of £20,000/QALY and £30,000/QALY, HD-TIV was estimated to be cost-effective at £23.75 and £30.70 per dose, respectively	HD-TIV resulted in significant benefits across adults ≥ 65 years of age and has the potential to be cost-effective vs. TIV. Results were most sensitive to the rVE of HD-TIV vs. TIV against hospitalizations	Yes (Sanofi)
Largeron N, et al., 2018 [70]	Australia	HD-TIV vs. QIV	Static decision tree model	Payer	Cost time horizon = 1 year Effect time horizon = 1 year	QIV AUS$9 Hospitalizations Medical visits Healthcare costs Deaths	2018, AUS$	rVE HD-TIV vs. TIV 24.2% * from FIM12 RCT [50] VE TIV 58.4% VE QIV 59.8% (based on prior CEA [100])	Costs 5% Outcomes 5%	DSA	In an average season, HD-TIV rather than QIV could prevent: 11,364 confirmed influenza cases, 17,576 cardiorespiratory-related hospitalizations, and 446 influenza-related deaths	HD-TIV vs. QIV in elderly adults ≥ 65 years of age is cost-effective at prices up to AUS$92/dose. HD-TIV becomes cost-saving if the price/dose does not exceed AUS$58	Yes (Sanofi)
Shireman T, et al., 2019 [101]	USA	HD-TIV vs. TIV	Cost–benefit analysis, person-level study	Payer (Medicare)	Cost time horizon = one influenza season Effect time horizon = one influenza season	HD-TIV $31.82 TIV $12.04 (CMS price schedule) Medical visits Hospitalization Home/hospice care Medications Vaccine price NR Skilled nursing facility Outpatient rehab	NR, USD$	NR	NR	Down-weighting top 1% of outliers	The $20 incremental cost of HD-TIV to TIV offset adjusted expenditures for a net benefit of $526 per nursing home resident and a financial return on investment of 27:1	HD-TIV reduced hospitalizations and resulted in lower Medicare expenditures. The magnitude of the estimated savings overwhelmed the incremental cost of HD-TIV vs. TIV	Yes (Sanofi)
Basile M, et al., 2020 [71]	Italy	HD-QIV vs. QIV	Static decision tree model	Healthcare system	1 year Deaths: life-year	Influenza cases Hospitalizations GP consultation ED visits Comedications Deaths Ex-factory vaccine price	NR, € Euro	rVE HD-QIV to QIV 24.2% * from FIM12 RCT [50]	Outcomes 3%	DSA	HD-QIV generated an excess 18,052 life years saved and 17,100 QALYs vs. QIV, saving €21.0 million to the healthcare system HD-QIV dominated QIV	HD-QIV could reduce the public health burden of influenza-related complications, and be cost-saving or cost-effective vs. QIV	Yes (Sanofi)
Borges M, et al., 2021 [72]	Portugal	HD-QIV vs. QIV	Decision tree model	NR	1 year	Influenza cases GP visits ER visits Hospitalizations Deaths Vaccine price NR	NR, € Euro	rVE HD-QIV to QIV 24.2% * from FIM12 RCT [50]	NR	DSA	HD-QIV reduced influenza cases by 12% and influenza-related deaths by 12%. HD-QIV reduced GP appointments by 1229 and ER visits by 532. Influenza-related hospitalizations were reduced by 10%. Respiratory hospitalizations were decreased by 14% and cardiorespiratory hospitalizations by 11%.	Switching to HD-QIV would contribute to reaching public health objectives, reducing excess mortality and the consumption of healthcare resources	Yes (Sanofi)
de Courville C, et al., 2021 [73]	Belgium	HD-QIV vs. QIV	Static decision tree model	Payer	1 year Deaths: life-year	QIV €16.46 HD-QIV €43.04 (NIHDI official prices) Influenza cases GP visits ER visits Hospitalizations Deaths	NR, € Euro	rVE HD-QIV to QIV 24.2% * from FIM12 RCT [50] VE QIV: 50% (based on RCT [102])	Outcomes 1.5%	DSA, PSAF	HD-QIV vs. QIV ICER €1397/QALY. HD-QIV was cost-effective considering a WTP threshold of €35,000/QALY	Key drivers of model outcomes were efficacy against influenza-associated hospitalization for HD-QIV vs. QIV, acquisition costs, the cost of influenza-related hospitalization and hospitalization rates	Yes (Sanofi)
Zeevat F, et al., 2023 [103]	Netherlands	HD-QIV vs. QIV	NR	NR	One season	Hospitalizations (all, respiratory, and CV) Complications Vaccine price NR	NR	NR	NR	NR	HD-QIV usage rather than QIV could have averted 220 hospitalizations, avoiding an expenditure of €1,219,779. Expenditure of €841,531 (i.e., 69% of the total costs) is attributable to avoidance of CV hospitalizations.	Switching from QIV to HD-QIV comes with cost savings. Benefits come from avoided CV-related hospital admissions	No
Alvarez P, et al., 2023 [74]	Belgium, Finland, Portugal	HD-QIV vs. QIV	Decision tree model	Payer, NHS	1 year Deaths: life-year	Comedication Influenza cases GP visits ER visits Hospitalization Vaccine price NR	NR	rVE HD-QIV to QIV 24.2% * from FIM12 RCT [50]	Costs 0% Outcomes 1.5 to 4%	DSA, PSA	HD-QIV resulted in improved health outcomes (visits, hospitalizations, and deaths) vs. QIV HD-QIV vs. QIV ICER €1397/QALY Belgium ICER €9581/QALY Finland ICER €15,267/QALY Portugal	HD-QIV would contribute to a significant improvement in the prevention of influenza health outcomes while being cost-effective	Yes (Sanofi)

* rVE values input into models may be inferred across vaccine families (i.e., researchers assumed equivalent VE between aTIV and aQIV; researchers assumed equivalent VE between HD-TIV and HD-QIV). ^†^ Budgetary impact analysis is a distinct form of economic analysis from cost-effectiveness analysis. aQIV, adjuvanted quadrivalent influenza vaccine; aTIV, adjuvanted trivalent influenza vaccine; CDC, US Centers for Disease Control and Prevention; CE, cost-effectiveness; CEA, cost-effectiveness analysis; CMS, Centers for Medicare & Medicaid Services; CV, cardiovascular; DSA, deterministic sensitivity analysis; ED, emergency department; ER, emergency room; GP, general practitioner; HD-QIV, high-dose quadrivalent influenza vaccine; HD-TIV, high-dose trivalent influenza vaccine; ICER, incremental cost-effectiveness ratio; ICU, intensive care unit; ID-TIV, intradermal TIV; LCI, laboratory-confirmed influenza; NIHDI, National Institute for Health and Disability Insurance; NIP, national immunization program; NHS, national health system; NR, not reported; PSA, probabilistic sensitivity analysis; QALY, quality-adjusted life year; QIV, quadrivalent influenza vaccine; QIVe, egg-based quadrivalent influenza vaccine; RCT, randomized controlled trial; rVE, relative vaccine effectiveness; RWE, real-world evidence; SEIR, susceptible, exposed, infected, and recovered; SIR, susceptible-infectious-recovered/protected/removed; TIV, trivalent influenza vaccine; TIVe, egg-based trivalent influenza vaccine; VAT, value-added tax; VE, vaccine effectiveness; WTP, willingness to pay.

CEA comparing enhanced vaccines with standard-dose vaccines estimated that enhanced vaccines were cost-effective in individuals ≥ 65 years of age. aTIV/aQIV and HD-TIV/HD-QIV were cost-effective compared with TIV/QIV, independent of setting, model design, perspective, rVE estimate, or acquisition cost difference (Table 2A,B).

### 3.2. Comparison between Enhanced Vaccines in CEA

CEA results were inconsistent when enhanced vaccines were compared with each other. Six studies compared aTIV/aQIV with HD-TIV/HD-QIV (mostly Seqirus-sponsored), ten studies compared HD-TIV/HD-QIV with aTIV/aQIV (mostly Sanofi-sponsored), and two studies compared QIVr with aQIV. Studies included static and dynamic designs, and perspectives ranged between healthcare system, societal, and third-party payer. Time horizons varied between one and multiple seasons. Discounting ranged from 0–5% for outcomes and costs. Most studies included deterministic and probabilistic sensitivity analyses. Findings remained robust across sensitivity analyses. Rate of hospitalization, rVE, and vaccine acquisition price were drivers of cost-effectiveness (CE) in many models (Table 3).

rVE inputs varied across studies. Most CEA comparing aTIV/aQIV versus HD-TIV/HD-QIV included direct estimates of rVE based on meta-analyses findings (Table 3A). On the other hand, CEA comparing HD-TIV/HD-QIV versus aTIV/aQIV often took an indirect approach, wherein a common comparator of TIV/QIV was used. The rVE estimate of 24.2% was commonly used for HD-TIV/HD-QIV versus TIV/QIV, based on findings from the FIM12 RCT [50] (Figure 1), whereas rVE ranging from 0% to 6% were used for aTIV/aQIV versus TIV/QIV (Table 3B; Figure 2).

Two CEA studies of interest were identified for QIVr (Table 3C). The first estimated the effect of switching from QIV/aQIV to QIVr in two age cohorts (≥18 years of age and ≥65 years of age) in the Spanish population using a static decision tree model [104]. The study estimated that mortality, hospitalizations, general practitioner visits, and emergency room services would decrease by 12%, 13%, 11%, and 12%, respectively, should the switch from QIV/aQIV to QIVr be implemented [104]. The second study did not find QIVr cost-effective compared with aQIV for individuals ≥ 65 years of age living in Spain. To achieve an incremental cost-effectiveness ratio (ICER) within the willingness-to-pay threshold, the rVE of QIVr versus aQIV would need to reach 34.1% [105].

**Table 3:** Cost-effectiveness studies evaluating adjuvanted vaccines versus high-dose vaccines (**A**), high-dose vaccines versus adjuvanted vaccines (**B**), and recombinant vaccine versus other enhanced vaccines (**C**).

Author Year	Country	Strategy	Model Type	Perspective	Time Horizon	Selected Costs	Year, Currency	rVE *	Discounting	Uncertainty Analysis	Findings	Author Conclusion	Industry Sponsor
**(A) Adjuvanted vaccines vs. high-dose vaccines**													
Nguyen VH, et al., 2021 [57]	France	aQIV vs. QIVe aQIV vs. HD-QIV	Static decision tree model	Payer	NR	QIV €11.11 aQIV €26.00 HD-QIV €26.00 (origin not specified) Healthcare visit In/outpatient complications Hospitalization Mortality	NR, Euro €	rVE aTIV vs. QIV 13.7% (95% CI 3.1, 24.2) * rVE aTIV vs. HD-TIV 3.2% (−2.5, 8.9) * rVE aTIV vs. TIV 13.9% (4.2, 23.5) * (from meta-analysis [92])	NR	DSA	Replacing QIVe with aQIV over a 3-year period can prevent: 56,028 influenza cases, 13,449 medical care visits, 30,815 outpatient complications, 3902 inpatient complications, and 745 influenza-associated deaths Budget savings were driven by avoidance of medical care visits costs (€470 K); outpatient complication costs (€788 K) and inpatient complication costs (€23.2 M)	aQIV for the older adult population would be clinically favorable, with a small incremental cost impact (Data for aQIV vs. HD-QIV not presented)	Yes (Seqirus)
Kohli MA, et al., 2021 [63]	UK	aQIV vs. HD-QIV	SEIR compartmental transmission model	Societal, National Healthcare Service	10 seasons	aQIV £11.88 HD-QIV £20.00 (list price) Hospitalization Vaccine Death Medical visits Complications	NR, GBP£	rVE aQIV vs. HD-QIV 3.2% (−2.5, 8.9) * (from meta-analysis [92])	Costs 3.5% Outcomes 3.5%	Scenario analyses	For ICER to fall below £20,000/QALY, unit price of HD-QIV should be less than £12.94, £10.44, or £7.67 for rVEs of −2.5%, 3.2%, and 8.9%, respectively aQIV is cost-saving vs. HD-QIV priced at the existing list price of HD-TIV	As the effectiveness of the vaccines was not statistically significantly different, the differences between the vaccines in clinical cases and influenza treatment costs are minimal	Yes (Seqirus)
Kohli M, et al., 2022 [58]	Germany	aQIV vs. QIVe aQIV vs. HD-QIV	SEIR compartmental model calibrated to German population	Societal, Statutory Health insurance	10 seasons from 2010–2019	QIVe €12.56 aQIV €19.21 HD-QIV €40.55 (reimbursed prices) Hospitalization Death In/outpatient visits Medication Sickness benefit Lost working time	NR, Euro €	aQIV vs. QIVe 13.9% (4.2, 23.5) * aQIV vs. HD-QIV 3.2% (−2.5, 8.9) * (from meta-analysis [92]) VE QIVe 62%, 24% and 79% against A/H1N1, A/H3N2 and B types (assumptions, related to meta-analysis [94] and systematic review [90])	Costs 3% Outcomes 3%	DSA, PSA	Both enhanced vaccines reduced the number of influenza cases, hospitalizations, and deaths in the German population compared with QIVe aQIV dominated HD-QIV because it was considered marginally more effective in the base case (rVE = 3.2%), and less costly to implement	aQIV may be cost-effective compared with QIVe at current prices. aQIV and HD-QIV had similar clinical effectiveness, but aQIV is less costly than HD-QIV The CE of aQIV was most sensitive to changes in VE and rate of hospitalization due to influenza	Yes (Seqirus)
Ruiz-Aragón J, et al., 2022 [64]	Spain	aQIV vs. HD-QIV	Static decision tree model Calibrated to the Spanish population	Societal, direct medical payer	Cost: three seasons Effect: lifetime	aQIV €23.00 HD-QIV €32.00 (list price) Hospitalization Death Medical visits Comedication Productivity loss	NR, Euro €	rVE aTIV vs. HD-TIV 4.0% (−0.05, 8.4) * (from meta-analysis published in own paper [64])	Costs 3% Outcomes 3%	DSA, PSA	Compared with HD-QIV, aQIV reduced: cases by 5405, primary care visits by 760, ER visits by 171, hospitalizations by 442, and deaths by 26 aQIV dominated HD-QIV, as it is less expensive and more effective from both the societal and direct medical payer perspectives	aQIV is a cost-effective vs. HD-QIV for older Spanish adults Vaccine costs are the most influential parameters in the model, followed by vaccine coverage	Yes (Seqirus)
Choi MJ, et al., 2022 [59]	South Korea	aQIV vs. QIV aQIV vs. HD-QIV	Static decision tree Analyzed across three age groups (65–74, 75–84, and ≥85 years of age)	Healthcare system	1 year	Hospitalization Death Complications Influenza cases Vaccine price	NR	aQIV vs. QIVe 13.9% (4.2, 23.5) * aQIV vs. HD-QIV 3.2% (−2.5, 8.9) * (from meta-analysis [92]) VE QIV 62%, 24%, and 63% vs. A(H1N1), A(H3N2), and B, respectively (from meta-analysis [94])	NR	DSA, PSA	Compared with QIV, aQIV reduced: cases by 35,390, complications by 1602, hospitalizations by 709, and deaths by 145 Compared with HD-QIV, aQIV reduced: cases by 7247, complications by 328, hospitalizations by 145, and deaths by 30	Replacing QIV with aQIV is predicted to reduce disease burden in the South Korean ≥ 65 years of age group Benefits of aQIV and HD-QIV are predicted to be similar due to comparable VE rVE was the most important factor influencing CE	Yes (Seqirus)
Jacob J, et al., 2023 [62]	Denmark, Norway, Sweden	aQIV vs. HD-QIV	Static decision tree model	Healthcare payer, societal	NR	QIV €9.10–11.00 aQIV 170–189% that of QIV HD-QIV €25 (public sources; assumption) Hospitalization GP visit Outpatient visit Comedication Lost productivity Death Complications Influenza cases	2022, Euro €	aQIV vs. HD-QIV 3.2% (−2.5, 8.9) * (from meta-analysis [92]) rVE HD-QIV to QIV 24.2% * from FIM12 RCT [50]	3–4% outcomes and costs	DSA, PSA	Across Denmark, Norway, and Sweden, aQIV vs. QIV could prevent a combined total of 18,772 symptomatic influenza infections, 925 hospitalizations, and 161 deaths in one influenza season across the three countries aQIV cost-saving vs. HD-QIV. As aQIV and HD-QIV were assumed to have comparable VE, the health benefits in favor of aQIV were marginal	Introducing aQIV to those ≥ 65 years of age may reduce the influenza disease and economic burden in Denmark, Norway, and Sweden	Yes (Seqirus)
**(B) High-dose vaccines vs. adjuvanted vaccines**													
Skinner L, et al., 2019 [106]	England and Wales	HD-TIV vs. aTIV	Static decision tree model	Public healthcare payer	1 year	Hospitalization Influenza complications GP consultations Death Vaccine list price	NR, GBP£	NR	Costs 0% Outcomes 3.5%	NR	HD-TIV vs. aTIV ICER £2154–8757/QALY for influenza/pneumonia hospitalizations analysis HD-TIV vs. aTIV ICER £2800 for respiratory hospitalizations analysis	HD-TIV is cost-effective vs. aTIV, driven by reduction in hospitalizations	Yes (Sanofi)
Basile M, et al., 2020 [71]	Italy	HD-QIV vs. aTIV	Decision tree model	Healthcare system	1 year Deaths: life-year	Influenza cases Hospitalizations GP consultation ED visits Comedications Deaths Ex-factory vaccine price	NR, Euro €	rVE HD-QIV to QIV 24.2% * from FIM12 RCT [50] rVE aTIV vs. TIV: 6.0% influenza cases (from retrospective cohort study of aTIV vs. virosomal-TIV [75]). No rVE sensitivity analysis stated.	Outcomes 3%	DSA	HD-QIV generated an excess 18,173 life years saved and 16,438 QALYs vs. aTIV HD-QIV vs. aTIV ICER €11,138/QALY	Vaccination with HD-QIV in those ≥ 65 years of age could be cost-effective vs. aTIV considering hospitalizations conditional on influenza cases	Yes (Sanofi)
Gibbons I, et al., 2020 [107]	England	HD-QIV vs. aTIV	Static decision tree model	Healthcare system	1 year	Influenza cases GP consultation Hospitalizations Deaths Vaccine price NR	NR, £GBP	NR, rVE HD-QIV vs. aTIV for three distinct analyses rVE from FIM12 RCT* [50] (specific value NR in abstract)	NR	DSA	HD-QIV was cost-neutral vaccination strategy (ICER: £824/QALY) vs. aTIV regarding influenza/pneumonia events in base-case scenario When hospitalizations were considered (broader respiratory and cardiovascular hospitalizations), HD-QIV dominated aTIV	HD-QIV could reduce the annual public health burden of influenza-related complications, while being a highly cost-effective, and in some cases dominant, alternative to aTIV in England Results remained robust across three values tested for the rVE of HD-QIV versus aTIV	Yes (Sanofi)
Net P, et al., 2021 [76]	USA	US standard of care with and without HD-TIV	Budget impact, decision tree framework	Medicare perspective	9 years	Influenza cases ED visits Hospitalizations Comedications Deaths Vaccine price NR	2019, US$	rVE HD-TIV vs. TIV 24.2% * from FIM12 RCT [50] rVE aTIV vs. TIV 0% (assumed 0% because no RCT data available). rVE varied to 4.7% aTIV vs. TIV in scenario analysis	0% costs NR outcomes	DSA, PSA	HD-TIV estimated to potentially avert 1,333,479 influenza cases, 769,476 medical visits, 40,004 ED presentations, 520,342 cardiorespiratory hospitalizations, and 73,689 deaths Generate $4.6 billion in savings over 10 years HD-TIV cost-saving under all the scenarios	HD-TIV provided improved efficacy and economic outcomes. Hospitalizations and rVE of HD-TIV vs. TIV were major cost drivers	Yes (Sanofi)
Rumi F, et al., 2021 [77]	Italy	HD-QIV vs. aQIV	Decision tree model	Health system	1 year	Hospitalizations GP visits ED visits Deaths Vaccine price NR	NR, Euro €	rVE HD-QIV to QIV 24.2% * from FIM12 RCT [50] rVE HD-QIV to QIV 18.2% in preventing CV hospitalization (from meta-analysis [108]) rVE aQIV vs. QIV 0% (assumed 0% because no RCT data available. Varied to 6% and 12% in scenario analysis)	NR	DSA, PSA	HD-QIV vs. aQIV ICER €7301/QALY rVE aQIV vs. QIV 0% ICER €9805/QALY rVE aQIV vs. QIV 6% ICER €14,733/QALY rVE aQIV vs. QIV 12% HD-QIV dominated aQIV, saving the healthcare system more than €53 million while improving clinical results	HD-QIV would be cost-effective when influenza hospitalizations were included, and cost-saving when the full burden of influenza is considered. DSA determined VE and rVE inputs most impactful on CE results	Yes (Sanofi)
Redondo E, et al., 2021 [78]	Spain	HD-QIV vs. aTIV	Decision tree model	Payer	6 months	Influenza cases GP visits ED visits Hospitalizations Deaths Vaccine price NR	NR, Euro €	HD-TIV vs. TIV 24.2% or 24.3% * from FIM12 RCT [50] rVE aTIV vs. TIV 6.0% influenza cases and hospitalizations (from retrospective cohort study of aTIV vs. virosomal-TIV [75]). Varied to 0.0% and 6.0% in sensitivity analysis	QALY 3%	PSA, DSA	Switching from aTIV to HD-QIV would prevent: 6476 cases of influenza, 5143 visits to the GP, 1054 visits to the ED, 9193 episodes of hospitalization due to influenza or pneumonia, and 357 deaths due to influenza HD-QIV vs. aTIV ICER €24,353/QALY	HD-QIV in people > 65 years of age is an influenza-prevention strategy that is at least cost-effective, if not dominant, in Spain.	Yes (Sanofi)
Nguyen VH, et al., 2022 [109]	Canada	QIVe vs. 1. QIVe + aTIV 2. QIVe + HD-QIV 3. QIVc + aTIV	SEIR model	Health care system	8 years	Hospitalization Death Medical visits Comedication Vaccine price NR	NR, Canada$	rVE QIVc vs. QIVe when egg-adapted against A/H3N2 15.6% (7, 20) rVE HD-QIV or aTIV vs. QIVe when egg-adapted against A/H3N2 9% (7.2, 10) rVE HD-QIV or aTIV vs. QIVe when matched against A and B strains 24% (9.7, 36) (all calculated based on electronic medical records [110])	5%	DSA, PSA	Three scenarios were compared vs. baseline scenario of QIVe for all age groups Scenario 1 (QIVe + aTIV for adults ≥ 65 years of age) was cost-saving Scenario 2 (QIVe + HD-QIV for adults ≥ 65 years of age) was above willingness-to-pay threshold at all rVE estimates Scenario 3 (QIVc + aTIV for adults ≥ 65 years of age) was cost-effective across all three rVE estimates, with ICER CA$1300 to CA$6900	Vaccination of individuals 6 months to 64 years of age with QIVc and ≥65 years of age with aTIV is cost-effective across varying assumptions of rVE and varying egg-adapted influenza seasons	Yes (Seqirus)
Mattock R, et al., 2021 [79]	England and Wales	HD-TIV vs. aTIV	Decision tree model	Healthcare payer	Cost: one season Effect: lifetime	aTIV £9.79 HD-TIV £20.00 (list prices) LCI cases that could result in a GP visit Hospital stays that could lead to premature death Vaccine price NR	2018, GBP£	rVE HD-TIV 24.2% or 24.3% * from FIM12 RCT [50] rVE aTIV vs. HD-TIV 0% LCI (assumed 0% because no RCT data available; varied to 6% and 12% in scenario analysis) rVE aTIV vs. HD-TIV 0% hospitalization (estimated at 0% because no RCT data available; varied to 10% and 20% in scenario analysis)	Costs 0% Outcomes 3.5%	DSA	HD-TIV cost-effective vs. aTIV for all three hospitalization effectiveness scenarios, with ICER equal to £1932, £4181, and £8767 per QALY	HD-TIV is cost-effective vs. aTIV in people ≥ 65 years of age in England and Wales DSA identified the rVE of HD-TIV on hospitalization outcomes as an important area of uncertainty	Yes (Sanofi)
Drago G, et al., 2020 [80]	Spain	HD-QIV vs. aTIV	Decision tree model	Healthcare system	Cost: 1 year Effect: lifetime	Influenza cases Hospitalizations GP consultation ED visits Deaths Vaccine price NR	NR, Euro €	rVE HD-TIV 24.2 * from FIM12 RCT [50] rVE aTIV vs. TIV 6.0% influenza cases (from retrospective cohort study of aTIV vs. virosomal-TIV [75]). Varied to 0% and 6% in sensitivity analysis	Outcomes 3%	DSA	Compared with aTIV, HD-QIV generated an excess 3514 life-years and 3304 QALYs, resulting in an ICER of €23,872/QALY	HD-QIV could annually reduce the public health burden of influenza-related complications and be cost-effective in influenza vs. aTIV VE against influenza cases and rVE against influenza and pneumonia hospitalizations were the most impactful parameters in DSA	Yes (Sanofi)
van Aalst R, et al. 2021 [81]	USA	HD-TIV vs. aTIV	PERR method	Healthcare payer	NR	HD-TIV $46.23 aTIV $48.26 (average list price) Hospitalization Vaccine price NR	NR, USD$	rVE HD-TIV vs. aTIV 7% (2.3, 12) respiratory or CV hospitalization; 12% (3.3, 20) respiratory hospitalization (from retrospective cohort study [111])	Costs NR Outcomes NR	PERR	Hospitalization rates for respiratory disease in HD-TIV and aTIV recipients were 187 and 212 per 10,000 persons-years, respectively. Estimated net savings of HD-TIV were $34 ($10–$62) per recipient	HD-TIV was associated with lower hospitalization costs vs. aTIV. HD-TIV remained cost-saving in all sensitivity analyses performed for hospitalizations with underlying cardiorespiratory disease	Yes (Sanofi)
**(C) Recombinant vaccine versus other enhanced vaccines**													
Drago Manchón G, et al., 2021 [104]	Spain	Switching from QIV/aQIV to QIVr	Decision tree model	Spanish National Healthcare System	1 year	Influenza cases GP visits ER visits Hospitalizations Deaths Vaccine price NR	NR	VE QIV 50% influenza cases (based on RCT [102]) VE QIV 40% influenza hospitalizations (from meta-analysis [112]) rVE QIVr vs. QIV 30% (from RCT [113]) rVE aQIV vs. QIV 6% (from retrospective cohort study of aTIV vs. virosomal-TIV [75])	NR	NR	Mortality, hospitalizations, GP visits, and ER services would decrease by 12%, 13%, 11%, and 12%, respectively, should the switch from QIV (and from aQIV for those ≥ 65 years of age) to QIVr be implemented	Costs, currency year, discounting, and uncertainty analyses could not be assessed	NR
Ruiz-Aragón J & Márquez-Peláez S 2023 [105]	Spain	QIVr vs. aQIV	Static, decision tree model	Public payer, societal	1 year	aQIV €13 QIVr €25 (list prices) Influenza cases Hospitalizations GP consultation ED visits Deaths	2021, Euro €	rVE QIVr vs. aTIV 10.7% (2.7, 17.9) inpatient stays (from observational study [114])	Costs 3% Outcomes 3%	PSA, DSA	QIVr vs. aQIV ICER €101,612.41/QALY To be cost-effective, rVE of QIVr vs. aQIV would need to be 34.1%	QIVr is not cost-effective vs. aQIV for older persons living in Spain	Yes (Seqirus)

* rVE values input into models may be inferred across vaccine families (i.e., researchers assumed equivalent VE between aTIV and aQIV; researchers assumed equivalent VE between HD-TIV and HD-QIV). aQIV, adjuvanted quadrivalent influenza vaccine; aTIV, adjuvanted trivalent influenza vaccine; CE, cost-effectiveness; CV, cardiovascular; DSA, deterministic sensitivity analysis; ED, emergency department; ER, emergency room; GP, general practitioner; HD-QIV, high-dose quadrivalent influenza vaccine; HD-TIV, high-dose trivalent influenza vaccine; ICER, incremental cost-effectiveness ratio; LCI, laboratory-confirmed influenza; NR, not reported; PERR, prior event rate ratio; PSA, probabilistic sensitivity analysis; QALY, quality-adjusted life year; QIV, quadrivalent influenza vaccine; QIVe, egg-based quadrivalent influenza vaccine; QIVr, recombinant quadrivalent influenza vaccine; RCT, randomized controlled trial; rVE, relative vaccine effectiveness; SEIR, susceptible, exposed, infected, and recovered; TIV, trivalent influenza vaccine; TIVe, egg-based trivalent influenza vaccine; VE, vaccine effectiveness.

### 3.3. Systematic Reviews of CEA

Further to primary CEA studies, several systematic reviews of CEA for enhanced vaccines in older adults have been published [31,32,33,34]. A systematic review of the cost-effectiveness of HD-TIV in individuals ≥ 65 years of age identified that HD-TIV was either cost-effective or cost-saving across multiple analyses [33], and that the prevention of cardiorespiratory complications was a potential driver of economic benefits [33]. Many of the studies included in this systematic review were also included in our analysis (such as [65,66,67,68,101], which are included in Table 2B). A comprehensive review from Canada suggested that aTIV, HD-TIV, and QIV were cost-effective compared with TIV for individuals ≥ 65 years of age, but noted a lack of head-to-head comparisons between QIV, HD-TIV, and aTIV [31]. The authors suggested that future studies should include real-world evaluations, and that methodological, structural, and parameter uncertainty should be assessed in CEA [31]. Similarly, a systematic review of seasonal influenza vaccine economic evaluations in individuals ≥ 60 or ≥65 years of age from the European Union recommended linking economic evaluations to observational cohort studies, RCTs, or other long-term, prospective, controlled studies [32]. The authors pointed out the need for data over multiple seasons, owing to influenza virus mutations and the potential for vaccine mismatch [32]. Finally, a review of economic analyses of aTIV in older adults identified aTIV as cost-effective or cost-saving compared with no vaccination or non-adjuvanted vaccines [34].

## 4. Critical Assessment of CEA Inputs and Approaches

CEA is a robust process that involves a variety of inputs, including, but not limited to, price, effectiveness, and utility, which supports decision analysis and is amenable to sensitivity testing [115]. Many economic analyses are performed to a high standard in accordance with gold-standard reporting guidelines for CEA, such as Consolidated Health Economic Evaluation Reporting Standards (CHEERS) 2022 [116]. Selection of robust inputs is of critical importance to the usability of findings from CEA models.

### 4.1. Effectiveness Input

#### 4.1.1. Importance of RWE for Influenza

It is important for public health officials to closely monitor circulating virus strains and for annual influenza vaccines to be adjusted and assessed on a seasonal basis [7]. Although vaccinated individuals achieve a level of cross-protection during mismatched seasons, VE usually decreases during mismatched seasons [90], and other factors, such as prior exposure, timing of vaccination, and waning immunity, may affect VE. The ability to assess vaccine performance in real time over multiple seasons, including those characterized by antigenic mismatch [8], is of high value for influenza.

Whereas RCTs aim to answer a focused research question by minimizing bias and confounders through randomization, blinding, and patient selection criteria, observational studies better reflect real-world conditions and are more easily performed over multiple influenza seasons with different circulating strains. Studies of real-world data sources may evaluate larger, more diverse, and more representative study populations than RCTs, potentially leading to more generalizable and clinically relevant results [27,28]. RWE may be used more often for influenza vaccine recommendations than for other vaccines or decisions in other disease areas [117,118], owing to timeline, cost, ethical, and enrollment difficulties of conducting RCTs to evaluate influenza vaccines in older individuals [28,119]. However, RWE may be subject to bias and similar studies may return conflicting results. For example, as assessed by Gärtner et al., 2022, of the seven retrospective cohort studies included in a systematic review discussing RWE of enhanced vaccines for older adults, three were found to have serious risk of bias owing to ‘inadequate control for important confounders’, ‘selection of reported outcome’, and ‘selection of participants’, and four were at moderate risk of bias [11]. RCTs themselves may also be subject to selection and/or informational bias, and new ways of defining ‘high-quality evidence’ have been proposed [120].

Multiple tools are used to assess and describe the risk of bias in non-randomized studies, and these approaches are very important for assessing the quality of RWE. Meta-analyses and systematic reviews may assess the risk of bias between studies (e.g., using Egger’s test to assess potential positive publication bias) or within studies (e.g., using the GRACE, Cochrane Risk of Bias, ROBINS-I, or AMSTAR 2 tools) to rank study design, conduct, and evidence against several parameters to determine an overall risk of bias for individual studies [11,92,121,122]. To support the transparent communication of findings, the structured template and reporting tool for real-world evidence (STaRT-RWE) provides guidance endorsed by the International Society of Pharmacoepidemiology and the Transparency Initiative [123]. STaRT-RWE aims to support researchers by setting clear reporting expectations, leading to reduced misinterpretation and improved validity assessment [123]. A review of RWE studies published using this template shows that STaRT-RWE has the potential to improve the reporting standards for RWE studies [124].

From a public health perspective, policymakers should understand epidemiological methods and have familiarity with seasonal influenza patterns to utilize RWE studies appropriately for decision-making. Confounding factors, such as comorbidities, health status, or previous history of vaccination, can alter estimates of effectiveness in studies without randomized designs [119]. In observational studies, different methods to identify and adjust for confounding factors can be used, including multivariate sensitivity analysis, restriction, matching, and stratification [119]. Early enhanced vaccine RWE studies in Italy, including Mannino et al., 2012, determined that aTIV reduces the risk of influenza- or pneumonia-related hospitalization by 25% compared with TIV in older adults [85]. This study used a prospective, observational design to capture evidence from multiple influenza seasons between 2006–2009, and stratification and statistical procedures to control for confounding, such as propensity-score-based multivariate analysis [85]. In this case, bias inherent in the non-randomized design may have diminished the impact of effectiveness findings (i.e., bias towards the null, as the authors suggest that their estimate may have under-reported the number of influenza- or pneumonia-related hospitalizations prevented by aTIV compared with TIV [85]. Bias towards the null arising from misclassification of outcomes has been mentioned in this and other studies of enhanced vaccines [85,125].

Use of real-world inputs in CEA is increasing as regulators and payers recognize the value of diverse measures and high-quality RWE in informing healthcare decision-making [30,126]. When selecting effectiveness inputs for use in CEA, there is a need for practicality, to ‘do the best with the available data’, and to continue to prioritize analyses of patient-centric endpoints (e.g., hospitalization) in the real-world setting. For example, a Dutch study found that a major driver of cost savings with enhanced vaccines compared with standard vaccines in older adults was the prevention of cardiovascular-related hospital admissions [103], a real-world endpoint that may not be practical to study in a RCT setting. Furthermore, the practical real-time use of RWE has been demonstrated during the coronavirus disease 2019 (COVID-19) pandemic, a setting in which rapid policy decisions were required to save lives [120,127,128]. RWE aided the characterization of COVID-19 natural history, symptoms, and identification of clinical features associated with increased disease severity [127,128]. Real-world data provided confidence in the effectiveness and safety of COVID-19 vaccination in special populations, such as pregnant women, who were excluded from vaccine clinical trials [129]. Although the authors pointed out that most of the RWE reviewed had some risk of bias, the available data were sufficient to be highly reassuring to patients and providers who had to make decisions based on available data at the time [129].

With increased influenza rates in 2022–2023 compared with pandemic years [130], and risk of co-infection with influenza in patients with COVID-19 [131], there is a clear need to prevent extra hospitalizations to maintain hospital bed capacity; adequate protection of older individuals from influenza with enhanced vaccines supports this goal.

#### 4.1.2. Importance of RWE Meta-Analysis

Although several systematic reviews of enhanced vaccines support the comparable effectiveness of aTIV/aQIV and HD-TIV/HD-QIV for older adults [11,92,132], in the absence of RCT data and head-to-head comparisons between enhanced vaccines, different approaches to model assumptions and evidence strength grading may explain some variation in CEA findings across studies and industry sponsors. The European Centre for Disease Prevention and Control (ECDC) 2020 technical report on the efficacy, effectiveness, and safety of newer and enhanced seasonal influenza vaccines determined that the evidence base for the efficacy/effectiveness of enhanced influenza vaccines is ‘limited’ and comparability of enhanced vaccines with traditional seasonal influenza vaccines is ‘uncertain’ because of a lack of literature and because of clinical and statistical heterogeneity [133]. In the report, using GRADE criteria, relative efficacy data with HD-TIV versus TIV from one RCT (rVE 24.2%) and relative efficacy data with QIVr versus QIV from another RCT (rVE 30%) were classified as moderate-strength evidence. Conversely, VE data from five observational studies across three seasons (2011–2012, 2017–2018, and 2018–2019; VE 44.9%) were graded as low-strength evidence, because the data were generated from non-randomized sources and subject to risk of bias and imprecision [133]. In this context, different rVE estimates have been used by different researchers in CEA to model the economic benefits of aTIV/aQIV compared with other options (Figure 1 and Figure 2).

Other systematic reviews highlight the limitations of available RCTs that evaluate enhanced vaccines [134] and the potential value in using rVE estimates from RWE (as well as from RCTs) for HD-TIV [135]. After publication of the ECDC report, Gärtner et al., 2022 found similar effectiveness between aTIV and HD-TIV in seven RWE studies, whereas aTIV was more effective than HD-TIV in three studies [11]. From a policy perspective, countries considering RWE when making vaccine recommendations have recommended aTIV/aQIV, alongside other enhanced vaccines, such as HD-TIV/HD-QIV, in individuals ≥ 65 years of age [18,19,20].

Best-available estimates of rVE may include those arising from systematic reviews, meta-analysis, and network meta-analyses [31], which enable comparison of three or more interventions simultaneously [136,137]. Meta-analyses of real-world data may provide more robust estimates of effectiveness based on pooled sources of evidence compared with those provided by single studies. Among composite studies of enhanced vaccines in older adults, meta-analyses by Domnich et al., 2022 and Coleman et al., 2021 showed that aTIV and HD-TIV provide comparable effectiveness, which is supported by the Gärtner et al., 2022 systematic review; Lee et al., 2021 showed that HD-TIV is more effective than TIV [11,92,132,135]. These analyses were performed across large patient populations with data from multiple influenza seasons. rVE estimates from meta-analysis sources have been used in several CEA models assessing enhanced vaccines (Figure 1) [57,58,59,60,62,63,64], and some studies have produced novel meta-analysis estimates for use as part of a CEA [60,64].

When head-to-head trials are not available and comparisons are needed across multiple vaccines, network meta-analysis (also known as mixed treatment comparisons or multiple treatments meta-analysis) is an additional methodological option that enables the effectiveness of three or more vaccines to be compared in a single statistical analysis to aid decision-making [136,137]. Existing studies of rVE between vaccine pairs are organized into a network linked by direct and indirect comparisons [136,137]. This approach enables comparative ranking between vaccines and, similar to traditional meta-analysis methods, may produce a more precise estimate of relative effectiveness than that estimated from single studies [136,137]. The utility of network meta-analyses to assess relative effectiveness has also been established for COVID-19 vaccines [138,139]; one network meta-analysis analyzing the relative effectiveness and safety of approved seasonal influenza vaccines in different age and patient risk groups has been published [140].

#### 4.1.3. Limitations of Currently Available Influenza RCT Evidence

The HD-TIV versus TIV rVE point estimate from the FIM12 RCT is used consistently in CEA of HD-TIV (Figure 1 and Figure 2). Although an important and well-designed study, the use of a single rVE estimate across multiple CEA may not reflect the reality of influenza, of which VE estimates may change seasonally because of virus mutations [32]. Use of the same efficacy or effectiveness data in multiple CEA may also over-represent a limited evidence base [32]. Variation in VE reflects the reality of changing vaccine performance across seasons and emphasizes the importance of continuous and current effectiveness data collection to underpin influenza vaccine policy.

A systematic review and meta-analysis of high-dose versus standard-dose influenza vaccine RCTs in adults ≥ 65 years of age illustrated the importance of understanding vaccine effects on influenza-associated hospitalizations and deaths, and these outcomes cannot be assessed from the high-dose influenza vaccine RCT evidence base [134]. Data from immunocompromised individuals were also lacking [134]; exclusion of high-risk populations has been identified as a general limitation of influenza vaccine RCTs [141]. The authors concluded that, even with RCT data comparing HD-TIV versus TIV, there is limited evidence confirming a reduction in LCI cases with HD-TIV, and limited evidence regarding clinically relevant outcomes [134]. The authors stated that longer-term pragmatic trials are needed to demonstrate impact in real-world settings [134].

More broadly, the limitations of RCT evidence have been highlighted by the pressing need for current evidence describing real-world endpoints during the COVID-19 pandemic [120]. RCTs may have practical, ethical, and timeline concerns; meta-analyses may also be affected by the inclusion of flawed individual RCTs that require subjective assessment of certain methodologies of constituent studies [120]. Conceptual proposals, such as next-generation evidence-based medicine (EBM), or EBM plus (EBM+), contend that taking a broader approach to defining clinically actionable evidence is necessary in certain situations, such as when information is needed for rapid and urgent decision-making [120,142]. Research groups have proposed new frameworks for evidence appraisal using interdisciplinary, pluralistic, patient-centric, and/or complex system paradigms to complement traditional hierarchical study design-driven approaches [120,142]. The COVID-19 pandemic has taught us that even without RCT evidence ‘we cannot do nothing’ [143].

### 4.2. Vaccine Acquisition Price

In CEA in which rVE estimates for aTIV/aQIV and HD-TIV/HD-QIV are comparable, vaccine acquisition price can be the major driver of CE estimates (Table 3). However, determining the price paid for vaccines is challenging, because vaccines are purchased from manufacturers with pricing subject to proprietary negotiation and rebates; some studies use adjustment methods to estimate vaccine acquisition and administration costs [62,144,145]. Furthermore, specific vaccine prices, or type of price (e.g., list, reimbursed price, etc.) are not always disclosed in CEA, which prevents robust comparative assessment.

### 4.3. Sensitivity/Scenario Analyses

Best practices in CEA call for interrogating model inputs and assumptions through one-way, multivariate, and probabilistic sensitivity and scenario analyses [30,146]. Varying model assumptions in a one-way or multivariate manner assists in identifying which parameters drive ICERs; these are often illustrated within tornado plots. Estimates from composite probabilistic sensitivity findings indicate how often ICERs may sit within willingness-to-pay thresholds; for example, when multiple parameters are randomly varied simultaneously across pre-set ranges, often illustrated on a cost-effectiveness plane. As public health authorities make recommendations that often remain in place for years before re-appraisal, decision-making incorporating assessment of the most extreme scenarios from CEA is of sound public interest. Furthermore, for infectious disease modeling, such as influenza, more methodologically complex dynamic models are valuable [31] because they are able to incorporate varying disease state disutility inputs, the likelihood of transition between different disease states, and the likely duration of disease states for a hypothetical cohort of individuals.

Many CEA of enhanced influenza vaccines account for aspects of parameter uncertainty (e.g., variance of rVE), although measures taken to assess methodological uncertainty (e.g., discount rates and time horizon) and structural certainty (e.g., static or dynamic models) were more difficult to assess. rVE is often varied in sensitivity/scenario analyses and identified as a key driver influencing cost-effectiveness estimates. Other CEA vary parameters not limited to vaccine coverage rate, VE at baseline, hospitalization rates, case fatality rates, outpatient complications, baseline utility, vaccine acquisition price, human capital costs, and discount rates for costs and/or outcomes.

### 4.4. Interpretation of ICERs

It is difficult to compare ICERs across studies, particularly from analyses performed in different markets/countries; however, aTIV/aQIV and HD-TIV/HD-QIV are estimated as consistently cost-effective compared with TIV/QIV across countries (Table 2 and Table 3). Between CEA studies, estimated ICER estimates may differ. Not overlooking variations between markets, including differences in vaccines prices, costs of disease management, and opportunity costs, current thinking is that variations in ICERs are generally determined by two core drivers: vaccine acquisition price and rVE. From a practical perspective, despite differences in the rVE inputted into models, comparable rVE has been seen between enhanced vaccines from RWE [11,92,132]; thus, the fundamental driver of ICER differences may be vaccine acquisition price. Currently, adjuvanted vaccines are often priced less than high-dose vaccines.

## 5. Future Directions and Conclusions

### 5.1. Future Directions

As novel vaccine technologies become available, including nucleoside-modified messenger RNA vaccines [147], RWE-driven CEA for comparative assessment may become even more important. ‘Big data’ may be a valuable source of RWE as datasets become more analyzable, particularly when these data allow for alignment with patient-centric EMB+ approaches [120,142]. RCTs will not be replaced, but there is a need to rely more on RWE obtained from high-quality studies; as such, developing frameworks to define and/or rank RWE may have merit [29].

The continuous development of CEA models that account for the uncertainty of influenza in future seasons relies on updated RWE and robust use of sensitivity analyses. Effectiveness values from across multiple seasons allow for policymakers to consider more realistic and representative estimates accrued over time. In traditional evidence hierarchies, RWE may be graded as lower strength than RCT data because retrospective and observational studies contain bias [120]; however, RWE is particularly important to assess for influenza. Recent lessons from COVID-19 pandemic responses have illustrated how RWE can guide rapid public health action [120]. Network meta-analyses, especially those with value-of-information analysis, may become best practice sources for effectiveness inputs. Increased understanding of methods to control bias in real-world studies, and frameworks to enhance transparency in RWE publications, may make RWE an increasingly more acceptable contributing data source for vaccine policymakers.

Influenza B, a more genetically stable virus than influenza A, becomes the predominant strain compared with influenza A approximately every 4–5 years and is generally perceived to lead to milder disease than influenza A [148]. Outcomes data have challenged this perception, with some studies finding similar or excess mortality associated with influenza B as compared with influenza A [148]. QIVs that protect against influenza B have achieved lower effectiveness rates than anticipated, suggesting that more study of influenza B is required [148]. Future RWE studies may support preparedness against future changes in the relative prevalence and impact of influenza A and B.

Secondary bacterial infections may account for a substantial proportion of influenza-related mortality during pandemics [149]. The most common co-infection pathogens include *Streptococcus pneumoniae*, *Staphylococcus aureus*, *Streptococcus pyogenes*, and *Haemophilus influenzae* [149]. The impact of influenza vaccination against secondary bacterial infections, or even in full, has not been widely studied clinically, but evidence suggests a protective effect against mortality outcomes related to invasive secondary disease [149]. Devising methods to identify and capture the value of potential protection against invasive bacterial disease within influenza vaccine CEA may allow for a more accurate representation of the value of influenza vaccines.

### 5.2. Conclusions

Across many studies, aTIV/aQIV and HD-TIV/HD-QIV demonstrate cost-effectiveness against TIV/QIV, despite diversity in model type, vaccine acquisition price, rVE estimate, and study perspective in individuals ≥ 65 years of age. aTIV demonstrates similar rVE compared with other enhanced vaccines across multiple influenza-related outcomes in older adults based on RWE.

Despite the bias inherent in their design, RWE studies provide crucial information needed in CEA. Sensitivity analyses within CEA are important to identify which parameters present greatest uncertainty, while probabilistic sensitivity analyses can provide an overall view of the robustness of output estimates. Well-constructed meta-analyses may reduce uncertainty regarding individual rVE point estimates and provide the best estimates of rVE. Although many variables are included in influenza vaccine CEA, rVE and vaccine acquisition price are key drivers of ICERs. In most markets, adjuvanted vaccines are priced lower than high-dose vaccines.

Overall, data from RWE and CEA provide clinical and economic rationales for the use of enhanced vaccines, such as aTIV/aQIV, in people ≥ 65 years of age. In addition to price considerations, countries that consider RWE when making vaccine recommendations have preferentially recommended aTIV/aQIV, HD-TIV/HD-QIV, and/or QIVr, in individuals ≥ 65 years of age [18,19,20,21,22].

## Data Availability

Not applicable.
